# Life-course immunology: reframing sex differences in the immune system to better understand women’s health

**DOI:** 10.1186/s13293-026-00889-7

**Published:** 2026-05-12

**Authors:** Syreen Goulmamine, Sarah Chew

**Affiliations:** Society for Women’s Health Research, Washington, DC USA

**Keywords:** Immunology, Immunity, Life-course, Immunity, Sex differences, Hormonal transitions, Sex as a biological variable, Women’s health

## Abstract

Sex differences are observed in the immune system in innate and adaptive immune responses. These sex differences can be pronounced during periods of significant hormonal change for women, such as puberty, pregnancy, and menopause. Despite evidence of these immunological sex differences shifting and changing during the life course, some research imprecisely conceptualizes sex differences as simply comparative. The dynamic nature of sex differences in the immune system requires moving beyond static male–female comparisons that ignore life-stage transitions and instead adopting a life-course lens that recognizes how immune function—particularly in women—is continuously reshaped across biological transitions. Evidence demonstrates meaningful sex differences across immune-mediated conditions, infection outcomes, and reactogenicity, yet critical gaps remain in the understanding of mechanism and life-course variation in these immune responses. To meaningfully advance research on sex differences in immunity, studies must explicitly and intentionally account for life-stage transitions, moving beyond static male–female comparisons that do not account for life-stage transitions. This shift requires the use of research designs that appropriately consider sex differences and life stage and that studies are powered for life-stage immune questions. Sex differences in immunity are continuously reshaped by and across the lifespan due to aging, hormonal changes, and social and environmental exposures. The field of immunology and immunology research must reconsider life stage as a core principle of sex differences. Failure to intentionally study sex differences across the life course in immunology leaves major gaps in our understanding of immune function in women across the life course and drive disparities, inefficiencies, and inaccuracies in research, treatment, and clinical care.

## Highlights

- Immunology and immunology research must reconsider life stage as a core principle of sex differences.
- Failure to account for the importance of sex differences across the life course in immunology research leaves a gap in the understanding of sex differences in immunity resulting in disparities, inefficiencies, and inaccuracies in research, treatment, and clinical care, particularly among women.
- To meaningfully advance research on sex differences in immunity, we must design studies that explicitly and intentionally account for life-stage transitions, moving beyond static sex comparisons of male versus female.

## Introduction

Sex differences have been observed across health, disease, and treatment outcomes. Specifically, in the immune system, sex is a biological variable that impacts immune function and responses. Sex differences in innate and adaptive immune responses are well-documented, although, gaps remain in the understanding of the mechanisms mediating these responses [1]. In women, these sex differences can be particularly pronounced during periods of significant hormonal change, such as puberty, pregnancy, and menopause, as well as influenced by genetic and environmental factors. Despite evidence of these immunological sex differences beginning in utero and continuing with aging, certain research continues to conceptualize sex differences as static, obscuring how immune function is dynamically reshaped across critical life-course transitions [2]. Overlooking the dynamic nature of sex differences in the immune system within research undermines the rigor, reproducibility, and translation of research to real-world populations, particularly women. In a global context, this deficiency limits the impact of research on immune-related conditions, which impose significant global burdens. Broadly, to address the global health burden and ultimately, achieve health and well-being (Sustainable Development Goal (SDG) 3), immunology must integrate sex differences across the life course as a foundational tenet of the field. Importantly, immune function evolves across the lifespan in both females and males through the interaction of genetics, hormones, environmental exposures, and aging processes. However, several major immune-modulating biological transitions, including pregnancy, lactation, and menopause, occur uniquely in females and have historically been understudied or excluded from research. As a result, overlooking life-stage transitions in immunology research has disproportionately limited the evidence base guiding women’s health and treatment decisions.

In this article, we will discuss how understanding immune differences requires moving beyond static sex comparisons that do not account for life-stage transitions toward a life-course lens, recognizing that women’s immune function is continuously reshaped across developmental and reproductive transitions, including puberty, pregnancy, lactation, menopause, and aging, through the interplay of hormones, sex chromosomes, environment, and social exposures.

## Impact of life stage and sex differences in immunity

Mechanisms of disease and generalizability of research often remain uncertain due to study design limitations not considering life-stage transitions. A robust body of evidence demonstrates meaningful sex differences across immunity and immune-mediated conditions, yet critical gaps remain in the understanding of mechanisms and life-course variation in these cases [2]. For example, autoimmune diseases disproportionately affect females, accounting for nearly 80% of autoimmune patients, with higher prevalence observed in many conditions; however, differences in severity, progression, and long-term outcomes vary substantially in females by disease type and life stage [3]. Similarly, infection outcomes reveal sex-specific patterns in susceptibility and severity that differ by pathogen, age, and environmental context [4]. In vaccination research, females often exhibit stronger antibody responses and higher rates of vaccine reactogenicity compared with males, but these differences are not universal and may be shaped by dosing strategies, timing, and baseline immunity across life stages [5]. Emerging data also suggest that immune aging trajectories diverge between sexes with significant influences via genetics, past infections, and lifestyle, challenging the one-size-fits-all models of immunosenescence [6]. Despite these insights, mechanistic explanations and generalizability remain uncertain, in part due to study designs that inadequately account for hormonal transitions and other critical life stage shifts that shape immune function.

## A life-course framework for immune differences in women

**Fig 1.**
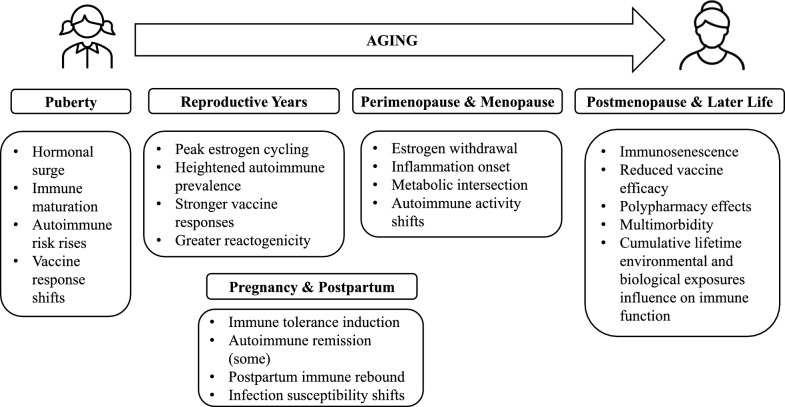
Life-stage transitions in women that influence immune function across the lifespan

Adopting a life-course immunology lens requires moving beyond static sex comparisons of male versus female and instead conceptualizing the influence of sex on immunity as dynamic and shifting across major biological transitions across the lifespan. As shown in Fig. 1, immune function in women is not fixed but evolves across the lifespan in response to hormonal changes, aging processes, metabolic transitions, and cumulative environmental exposures [7]. During puberty, hormonal shifts coincide with immune maturation and changing patterns of disease incidence. Pregnancy and the postpartum period represent profound immune recalibration and rebound unique to females, helping to explain fluctuations in autoimmune disease activity and infection risk for pregnant populations [8]. In perimenopause and menopause— life stages also unique to females— altered estrogen and progesterone levels intersect with inflammation, metabolic changes, and immune aging trajectories [9]. In later life, patterns of convergence or divergence between sexes are shaped by comorbidities, medication use, and lifetime exposures.

To meaningfully advance research on sex differences in immunity, we must design studies that explicitly and intentionally account for life-stage transitions, moving beyond static male–female comparisons. This means stratifying analyses by key biological phases, including puberty, pregnancy and postpartum, perimenopause and menopause, and later life, and incorporating measures of hormonal status, reproductive history, metabolic context, and cumulative environmental exposures. Applying this lens also helps investigators meaningfully implement the National Institutes of Health (NIH) Sex as a Biological Variable (SABV) policy, leading to more rigorous, efficient, and impactful research. Without embedding a life-course lens into study design, we risk generating findings that are incomplete, non-generalizable, and insufficient to inform precision prevention, vaccination strategies, and treatment across all populations.

## Future considerations

Historically, research has relied on male models and male data to study disease and treatments leaving major gaps in the understanding of women’s health. While sex differences in immunology are more well defined than in other fields, there remains a dearth of studies that incorporate a life-stage approach. The shift to a life-course model for immunology requires the use of research designs that emphasize sex differences research. In addition to meaningfully complying with the SABV policy for both publicly funded and privately funded research, investigators must ensure that both sex differences and life stage are appropriately considered in immunology research design and that studies are powered for life-stage immune questions. Further, research data must be appropriately analyzed and disaggregated. While pregnant and lactating populations have been historically excluded from clinical trials, consideration in recent years of including these populations and guidance from federal and academic leaders warrant reconsideration of this exclusion to better understand the impact of disease, treatments, and outcomes more broadly [10].

Importantly, adopting a life-course perspective does not require that every study examine all stages of the lifespan simultaneously. Rather, investigators can situate their work within a broader life-course framework by clearly defining the biological stage under investigation, reporting relevant contextual factors such as hormonal status or reproductive history when appropriate, and acknowledging how findings may differ across other life stages. Practical approaches include stratifying analyses by developmental or reproductive stage when feasible, incorporating measures of hormonal status or reproductive history, and designing complementary studies that collectively contribute to understanding immune processes across the lifespan.

## Conclusion

Sex differences in immunity are apparent in innate immunity, disease, outcomes, and treatment responses. These differences are continuously reshaped by and shifting across the lifespan due to aging, hormonal changes, and social and environmental exposures. Immune systems evolve across major biological transitions throughout life, particularly for women. With this understanding, the field of immunology and immunology research must reconsider life stage as a core principle of sex differences in women’s health.

The inability of the field to capture the importance of sex differences across the life course leaves a gap in the understanding of sex differences in immunity. Ultimately, this gap results in disparities, inefficiency, and inaccuracy in research, treatment, and care. If we want to achieve equality (SDG 5) in immunology, it is imperative to reconceptualize the understanding of sex differences in immunity. For men and women, proceeding through different biological milestones and hormonal changes can change immunity and treatment outcomes. Without studying or understanding these nuances, parity in research and care is unachievable. Further, in the aim to reduce the global burden of immune-related disease and more generally to reduce mortality from non-communicable diseases (SDG 3), it is critical to understand the mechanisms underlying immunity and the impact of lifespan on disease and disease outcomes. Without integrating a life-course understanding of sex differences in immunity, the field risks generating incomplete evidence that cannot adequately guide prevention, treatment, or clinical care across the populations it seeks to serve.

## Data Availability

Not applicable.

## Abbreviations

NIH: National institutes of health
SABV: Sex as a biological variable
SDG: Sustainable development goals
